# Perceptions of registered nurses on facilitators and barriers of implementing the AI-IoT-based healthcare pilot project for older adults during the COVID-19 pandemic in South Korea

**DOI:** 10.3389/fpubh.2023.1234626

**Published:** 2023-10-10

**Authors:** Sunjoo Boo, Hyunjin Oh

**Affiliations:** ^1^College of Nursing Research Institute of Nursing Science, Suwon, Republic of Korea; ^2^College of Nursing, Gachon University, Incheon, Republic of Korea

**Keywords:** AI·IoT-based healthcare, older adults, facilitators, barriers, digital literacy, public health

## Abstract

**Objective:**

This study explored the perceptions of registered nurses on the facilitators and barriers to implementing an AI/IoT (Artificial Intelligence/Internet of Things)-based healthcare pilot project, designed to prevent frailty and improve health behaviors by providing Bluetooth-enabled smart devices (including blood pressure and blood glucose meters) for the older adults aged over 65 years and above in South Korea.

**Methods:**

Using a qualitative descriptive methodology, interviews and qualitative surveys were conducted with 15 registered nurses from 11 public health centers. Data were analyzed using qualitative content analysis.

**Results:**

The study found that the AI·IoT-based healthcare pilot project was well received by participants, leading to increased client satisfaction and improved health behaviors. Government support and funding were crucial facilitators of project implementation. However, technical challenges and disparities in digital literacy among older adults pose significant barriers.

**Conclusion:**

The findings highlight the potential of AI·IoT technologies in improving the healthcare of older adults. Efforts to address technological challenges and enhance digital literacy among vulnerable populations are necessary for successfully implementing such interventions. Government support and ongoing training for healthcare professionals can help optimize the AI·IoT-based healthcare services for older adults.

## 1. Introduction

South Korea’s rapidly aging population and the ongoing COVID-19 pandemic have highlighted the need for innovative healthcare delivery methods (1). Among the approaches explored, the Artificial Intelligence/Internet of Things-based healthcare Pilot Project (AI·IoT-PP) launched by the Korean government in the latter half of 2020 stands out (1, 2). The AI·IoT-PP, designed to prevent frailty and improve health behaviors, provides Bluetooth-enabled smart devices such as blood pressure meters, blood glucose meters, smart scales, activity-tracking Bluetooth pedometers, and AI speakers to older adults aged 65 years and above (1). These devices are incorporated into the country’s home-visiting healthcare service, operated by registered nurses from public health centers (RN-PHC), to offer contactless public health services through a mobile health application (mHealth app).

The AI·IoT-PP initiative offers an integrated approach to remote healthcare consultations for older adults. In this method, participants undergo an initial health screening, after which they are categorized into different groups: healthy, high-risk frail, and frail. These categories determine the level and frequency of non-face-to-face health consultations. Each participant, based on their health conditions, is assigned specific health goals, fostering a proactive approach towards health maintenance. The Today’s Health app, pivotal to the AI·IoT-PP, allows for efficient data sharing between the AI·IoT devices and RN-PHCs, streamlining communication and collaboration.

The implementation of AI·IoT-PP is crucial in an era where face-to-face services have become limited due to the pandemic, particularly for vulnerable groups, including older adults, those from low-income families, and individuals with health problems (1, 3, 4). These challenges in providing public health services to vulnerable community members have underlined the importance of advancements in mHealth technologies (5). Notably, mHealth technologies can facilitate improved self-management, efficient home healthcare services, and enhanced communication and collaboration among older adults with chronic diseases (6).

The current proliferation of digital health initiatives, including AI·IoT-PP, presents new opportunities but also significant challenges, particularly in the context of an aging society and amidst public health emergencies like the COVID-19 pandemic (3, 6). While several studies have focused on the potential of digital health technologies, comprehensive research that specifically addresses the facilitators and barriers experienced by healthcare professionals in implementing these technologies remains scarce. Particularly in the context of home-visiting healthcare services, understanding these challenges is critical to the successful integration of AI·IoT technologies. Therefore, this study contributes to the existing body of knowledge by providing an in-depth analysis of RN-PHC’s experiences and identifies strategies for overcoming barriers and leveraging facilitators. The findings of this study have the potential to guide future healthcare policies and strategies for the implementation of digital health technologies, ultimately benefiting vulnerable groups and enhancing the overall quality of healthcare services.

In light of these considerations, this study aims to investigate the facilitators and barriers encountered by RN-PHC during the implementation of AI·IoT-PP in 2021. The primary focus is on understanding the implications of incorporating this new technology into home-visiting healthcare services. This study seeks to identify key facilitator and barrier domains and provide recommendations for improving the delivery and dissemination of AI·IoT healthcare services to older adults in public health. The study’s implementation-focused research question is: What are the facilitators and barriers related to AI·IoT-PP, as experienced by RN-PHC?

## 2. Materials and methods

### 2.1. Design

This study adopted a qualitative descriptive method (7, 8) to explore the perceptions of RN-PHC on their experience of facilitators and barriers while implementing AI·IoT-PP during the initial phase of the COVID-19 pandemic. The research employed a qualitative descriptive approach, as suggested by Sandelowski (7, 8), to gather and document narratives from managers. This method prioritizes proximity to the original data by employing straightforward sorting and coding techniques.

### 2.2. Participants and setting

The AI·IoT-PP, is a pilot initiative funded by the government of South Korea. This program involved 24 out of 256 public health centers across eight out of 17 cities and provinces, nationwide. The selection of participating public health centers for this government-run pilot health project was made after considering the project’s requisites and appropriateness within the predetermined budget constraints. The study focused on RN-PHC with a minimum of 2 years of experience in home-visiting healthcare services, and who had also engaged in the AI·IoT-PP for over 3 months. The study utilized a sample of 15 participants from 11 public health centers, with one to three participants per center (Table 1). To qualify for inclusion in the study, participants had to satisfy two criteria: (1) active involvement as a service provider in the AI·IoT-PP within the participating public health centers for over 3 months and (2) consented to permit the use of their data for the research objectives. The study did not define any specific exclusion criteria. Ethical approval was obtained from Ajou University medical center (AJOUIRB-SUR-2021-330).

**Table 1:** Demographic information (*n* = 15).

Participants	Sex	Region	Career (year)	Age	Interview
P1	F	Rural	14.6	56	Qualitative Survey
P 2	F	Rural	6.8	49	Qualitative Survey
P3	F	Urban	1.0	27	Qualitative Survey
P4	F	Urban	13.0	42	Qualitative Survey
P5	F	Urban	13.0	37	Qualitative Survey
P6	F	Rural	9.0	35	Qualitative Survey
P7	F	Urban	13.0	41	Qualitative Survey
P8	F	Rural	8.0	50	Qualitative Interview
P9	F	Rural	14.0	48	Qualitative Interview
P10	F	Rural	22.0	64	Qualitative Interview
P11	F	Rural	5.9	55	Qualitative Interview
P12	F	Urban	17.0	51	Qualitative Survey
P13	F	Rural	7.0	59	Qualitative Survey
P14	F	Rural	10.0	38	Qualitative Interview
P15	F	Rural	6.0	37	Qualitative Interview

### 2.3. Procedures and data collection

Data was gathered through a combination of individual interviews and qualitative surveys. Participants were given the option to select either a qualitative interview or a qualitative survey, taking into consideration their personal circumstances. The interview guide was developed through an extensive literature review on the adaptability of online-based public health projects (3, 9, 10). We used open-ended questions to encourage rich, detailed responses from the participants. Each interview lasted between 60–90 min. The interviews were carried out by the authors in the fall of 2021, either in a location chosen by the participant or online through the Zoom platform. All interviews were audio-recorded with the permission of the participants and later transcribed verbatim for analysis.

Table 2 included examples of interview questions: “Can you describe your experience with the AI·IoT-PP?,” “What are the advantages of contactless programs compared to face-to-face home healthcare services (in terms of health promotion for recipients, nursing practice for nurses, health center budgets, workforce, etc.)?,” “What do you consider as the barriers of AI·IoT-PP programs?,” and “What do you consider as the facilitators of AI·IoT-PP programs?”

**Table 2:** Interview guides.

What are the advantages of contactless programs compared to face-to-face home healthcare services (in terms of health promotion for recipients, nursing practice for nurses, health center budgets, workforce, etc.)? What do you consider as the barriers of AI·IoT-PP programs? What do you consider as the facilitators of AI·IoT-PP programs? Considerations and Improvements for Program Expansion Do you think the “AI·IoT-PP” can be applied to other public health centers with different conditions (population demographics, regional characteristics, etc.)? What aspects of the “AI·IoT-PP” need modification for program expansion, and how do you propose to make those modifications? What potential issues may arise when applying the program in other public health centers? How do you perceive the Today’s health app and smart devices used in the “AI·IoT-PP”?

AI·IoT-PP, artificial intelligence/internet of things-based healthcare pilot project.

In addition to the interviews, we employed a qualitative survey method that involved the distribution of open-ended questions via email (11). This approach was designed to give participants ample time and flexibility to provide thorough and in-depth responses, thereby reflecting their unique perspectives and experiences related to the research subject (11). The aim of this qualitative survey was to acquire a comprehensive and deep understanding of the phenomena being investigated.

### 2.4. Program

The goal of this initiative is to use AI·IoT technologies to deliver non-face-to-face healthcare consultations to older adults aged 65 years or older who experience difficulty in managing their own health, which not only improves the efficacy of home-visiting healthcare services but also assists in the provision of healthcare for the older adults in vulnerable groups, even during the COVID-19 pandemic. The program included initial health screening, tailored goal setting with the old adults, self-check of health using the Bluetooth device for 6 months, non-face-to-face health consulting using *Today’s Health app*, and health reevaluation at 6 months of enrollment. The *Today’s Health app* has been developed to automatically update the content of AI·IoT devices owned by older adults and share that information with RN-PHCs in an effort to enhance communication and collaboration.

Based on the initial health screening results, the older adults were divided into three groups: healthy, high-risk frailty, and frail. Every day, the participants measured their own blood pressure, blood sugar, and weight, using the provided health-measuring Bluetooth device and send the data to the *Today Health* app. Nurses in public health centers monitor the health status of participants in real-time via the *Today Health* app and provide health education and non-face-to-face health consultations to motivate participants to engage in healthy behaviors. The frequency and intensity of non-face-to-face health consultations vary among the groups. The frail group receives non-face-to-face health consultation and education twice a month, whereas the high-risk frail and healthy groups receive it once a month. All groups were assigned health related goals based on their health conditions, such as taking their medications on time every day, measuring their blood pressure daily, and walking for 30 min every day. The participants completed the missions and reported them on the app, while nurses checked whether they were completed. Incentives were provided according to how well the mission was accomplished.

### 2.5. Data analysis and rigor

We implemented both qualitative surveys and interviews in our data collection process, both adhering to the same interview protocols. The structured nature of the surveys ensured consistency across participants, while the open-ended interviews enabled the elicitation of deeper and more nuanced responses. This approach facilitated a comprehensive collection of data.

We utilized the qualitative data analysis method proposed by Hsieh and Shannon (12) for our research. The execution of this process was primarily carried out by one author (Oh) using ATLAS.ti 8 Windows software, resulting in a robust coding structure. The process began with discussing the definitions of codes and examining their similarities and differences until consensus was achieved by authors. These related codes were then categorized based on similar experiences. Subsequently, the interrelationships among the categories were analyzed, and initial themes were identified. The following step entailed group deliberations on the identified subthemes, paying special attention to ensure no crucial detail was missed, thereby bolstering the validity of the themes. After these collective discussions, the themes were refined and finalized, then defined and categorized, resulting in precise and substantial findings (Table 3).

**Table 3:** Data analysis.

Quotations	Primary codes	Subthemes	Themes
“The effect is greatly shown by improving interest in healthcare methods according to the use of smartphones and devices. Expectations for possible changes in health [-related] habits due to voluntary participation.” (1:14)	Use of Technology/Divices	Tech-empowered Health Autonomy	Facilitators: Digital Health Empowerment and Transformation
"People checked their blood pressure, blood sugar, steps, and weight, and could see how they compared to their old info. It was easy to manage their own health, and they could get into good habits with the daily missions. They looked at the numbers with the nurses, who could give advice without seeing them in person." (5:9)	Self-management		
“Once the older adults get used to it, being able to manage their health with their smartphones gives them a sense of accomplishment. A notable advantage is that they can see the results of their efforts. The pedometer was especially popular, although it had limited functionality, only measuring steps and pulse.” (10:16)	Health Monitoring		
	Real-time Health Advice		
	Sense of accomplishment		
	Voluntary Participation		
"The best part for the people doing the program was that doing the missions every day, kind of like homework, made their lives better by helping them exercise and manage their health." (8:12)	Health management/Improvement	Comprehensive Health Advancement through Structured Engagement	
"People were happy to take part because it was about their own health. The program included counseling about health and updates on their health indicators, and helped them learn how to take care of themselves. When they were given health info and rewards, they were more likely to keep participating in the program." (2:19)	Participant Engagement		
	Program Structure		
“During the COVID-19 pandemic, it was better to have fewer face-to-face visits and instead offer more remote services like phone counseling. This helped keep everyone safe.” (4:10)	Remote Health Services	Digitally Facilitated Personalized Health Management and Support	Facilitators: Government Support for Digital Health Management
“Because of the pandemic, managing your health became harder. But some people found it helpful that they could use technology to manage their health at home. For example, they could easily check their blood pressure and blood sugar levels and see how they were doing over time. (5:2)”	Use of Technology for Health monitoring		
“This program has enough budget to promote healthy behaviors and provide incentives for individualized health-related missions.” (5:19)	Financial Incentives		
	Support Beyond Material Provisions		
Users encountered situations where they couldn't fix issues with their device while trying to measure their health, so they had to ask for help. They felt let down when the step count disappeared due to machine errors after putting in effort to walk more. Moreover, they faced difficulty when their device did not connect well with their phone due to differences in phone model, which required them to manually input data, and that was quite annoying. (4:4)	Technical difficulties	Challenges in Digital Health Implementation and Participant Retention	Barriers: Tech Challenges in Digital Health for the older adults
Some participants dropped out of the project due to low participation, loss of contact, and other reasons, and there were difficulties because they did not return the devices. Despite my attempts to encourage them to complete the mission and stay in contact, I was unable to reach them, and their level of participation in the project was very low. Furthermore, there are no clear guidelines for withdrawing from the project. (5:12)	Participant Engagement		
	Program management Challenges		
“Some participants wanted to join the project, but they had little experience with the device, especially older and less tech-savvy individuals.” (1:23)			
“Based on the participants' characteristics, it seemed like there was a correlation between their economic status, education level, and ability to use mobile phones. Those who found it easy to use the device participated more actively and even took on additional missions. However, some participants found even the basic mission of wearing the activity tracker every morning challenging and dropped out of the project.” (6:12)			
It's taking a while for our participants to adapt to the devices. We found that there were only a few people who were suitable for the IOT project among the home visiting project participants. (9:2)			
In my opinion, we should aid vulnerable individuals. With solutions for mobile phone compatibility issues and adequate education, I think the IOT project can work for older adults and vulnerable participants (with help from their children if needed). (7:34)	Technological inexperience	Addressing barriers to technological adoption and adaptation among diverse populations	Barriers: The digital alienation of vulnerable older adults
	Socioeconomic and Educational Factors		
	Adaptation Challenges		
	Need for Assistance		

To enhance the credibility of our study, we initiated the analysis concurrently with data collection, which provided an immediate and continuous reflection on the data. We augmented the robustness of our findings through a cross-checking process with two other RN-PHCs. In addition, we adopted reflexivity and peer debriefing strategies. Reflexivity allowed us to continually assess our biases throughout the research process, while peer debriefing offered a platform for discussion with impartial peers, helping identify and correct potential biases. These strategies collectively fortified the credibility of our research. Regular meetings were held by the authors during the analysis phase to review the data, reflect on it, and discuss the results, further assuring the credibility and grounding of our findings (12, 13).

In order to meet our research goals and maintain a rigorous process, we adopted a clear methodological framework. This facilitated the capturing and recording of participants’ expressions using straightforward descriptive data (7, 8). We employed a comprehensive sampling strategy, enlisting participants from 11 out of 24 participating public health centers, which enabled us to encapsulate a spectrum of managerial perceptions regarding RN-PHCs.

## 3. Results

The RN-PHC expressed excitement about the potential benefits and possibilities of AI·IoT-PP. Despite identifying critical areas for improvement in the intervention and its implementation over the period of 6–9 months, they still gave the pilot program high evaluations. These insights are delineated below as key facilitators and barriers in the execution of the AI·IoT-PP. Furthermore, the AI·IOT initiative was viewed as an innovative healthcare approach and an expansion of public health services. Although remote public health services have been identified as a national priority, there are potential challenges concerning real-time interventions for the older adults who may be unfamiliar with digital devices. Consequently, these challenges need to be addressed, and the project has expanded.

### 3.1. Facilitators

The RN-PHC viewed the implementation of AI·IoT-PP as highly beneficial and pragmatic owing to several significant aspects.

#### 3.1.1. Digital health empowerment and transformation

Digital health technology has been transformative for older adults, encouraging proactive healthcare management through personal devices. Although these individuals initially faced challenges in using the technology, consistent training and support led to their proficiency, heightened satisfaction, and self-assuredness. They embraced the immediate feedback and self-monitoring capabilities offered by these tools, appreciating the accessibility and personalization. The introduction of mobile devices particularly saw high levels of satisfaction, illustrating the power of digital health empowerment and transformation in fostering health autonomy and advancing comprehensive health.

*The effect is greatly shown by improving interest in healthcare methods according to the use of smartphones and devices. Expectations for possible changes in health [-related] habits due to voluntary participation. (P1:14).*

*People checked their blood pressure, blood sugar, steps, and weight, and could see how they compared to their old info. It was easy to manage their own health, and they could get into good habits with the daily missions. They looked at the numbers with the nurses, who could give advice without seeing them in person. (P5:9).*

*Once the older adults get used to it, being able to manage their health with their smartphones gives them a sense of accomplishment. A notable advantage is that they can see the results of their efforts. The pedometer was especially popular, although it had limited functionality, only measuring steps and pulse. (P10:16).*

RN-PHCs underscore the effectiveness of a daily digital health program that fosters health autonomy and facilitates proactive self-care among participants. Central to this approach was a goal-setting functionality, viewed as indispensable for older adults to define and reach personal health objectives. Older adults were supported by diverse devices, incentives, and personalized health missions, all of which were integral to the sustained progress and success of the program. The ability to autonomously tailor and administer missions and rewards further underscored the program’s commitment to personalized care and highlighted the transformative potential of combining AI·IoT-PP in public health care.

*The best part for the people doing the program was that doing the missions every day, kind of like homework, made their lives better by helping them exercise and manage their health. (P8:12).*

*People were happy to take part because it was about their own health. The program included counseling about health and updates on their health indicators, and helped them learn how to take care of themselves. When they were given health info and rewards, they were more likely to keep participating in the program. (P2:19).*

#### 3.1.2. Government support for digital health management

During the COVID-19 pandemic, government support played a critical role in expanding digital health services to vulnerable groups, such as older adults. These efforts were made feasible through sufficient funding and capitalizing on South Korea’s widespread mobile technology use. Digital health tools provided valuable data, allowing healthcare professionals to enhance their counseling services and improve the precision and individuality of their interventions. Through the allocation of funds, the government effectively promoted healthy behaviors, provided incentives for health-related missions, and enabled access to crucial health devices, demonstrating its commitment to enhancing health outcomes in challenging times. This comprehensive approach to digital health management has led to high levels of participant satisfaction and the overall success of the program.

*During the COVID-19 pandemic, it was better to have fewer face-to-face visits and instead offer more remote services like phone counseling. This helped keep everyone safe. (P4:10).*

*Because of the pandemic, managing your health became harder. But some people found it helpful that they could use technology to manage their health at home. For example, they could easily check their blood pressure and blood sugar levels and see how they were doing over time. (P5:2).*

*This program has enough budget to promote healthy behaviors and provide incentives for individualized health-related missions. (P5:19).*

### 3.2. Barriers

The implementation of the project encountered notable challenges, which were primarily attributed to technical issues and substantial disparities in digital literacy skills among older adults. This disparity was particularly pronounced among economically and educationally vulnerable seniors who exhibited lower levels of digital literacy and were at a higher risk of facing digital exclusion.

#### 3.2.1. Tech challenges in digital health for the older adults

The adoption of digital health technologies by older adults has been marked by numerous technical challenges, spanning device malfunctions, software usability issues, and connectivity difficulties. A common problem is that devices like pedometers frequently disconnect or malfunction, which is particularly challenging given the older participants’ limited familiarity with technologies like Bluetooth. Additionally, delays in notification about these issues further complicate the user experience and pose obstacles during phone consultations. Therefore, it’s clear that additional tech support and resources are essential to address these challenges and ensure the successful integration of digital health technologies among the older population.

*Users encountered situations where they could not fix issues with their device while trying to measure their health, so they had to ask for help. They felt let down when the step count disappeared due to machine errors after putting in effort to walk more. Moreover, they faced difficulty when their device did not connect well with their phone due to differences in phone model, which required them to manually input data, and that was quite annoying. (P4:4).*

RN-PHC faced challenges in sustaining older adults’ participation in the digital health program due to varied interest and skill levels. Complications arose from dropouts not returning devices and the absence of clear withdrawal guidelines. These issues underscore the need for additional resources and strategic planning for effective implementation of digital health initiatives for the older population.

*Some participants dropped out of the project due to low participation, loss of contact, and other reasons, and there were difficulties because they did not return the devices. Despite my attempts to encourage them to complete the mission and stay in contact, I was unable to reach them, and their level of participation in the project was very low. Furthermore, there are no clear guidelines for withdrawing from the project. (P5:12).*

#### 3.2.2. The digital alienation of vulnerable older adults

Among older adults and vulnerable populations, such as those residing alone, certain individuals faced obstacles in participating because of the limited functionality of their mobile phones. Older adults or diminished cognitive abilities also encounter difficulties with device connectivity and other technical complications. Furthermore, a considerable number of individuals did not possess smartphones, which complicates their involvement. Particularly, the older adults required extensive education, encouragement, and assistance to actively engage in the project.

*Some participants wanted to join the project, but they had little experience with the device, especially older and less tech-savvy individuals. (P1:23).*

*Based on the participants’ characteristics, it seemed like there was a correlation between their economic status, education level, and ability to use mobile phones. Those who found it easy to use the device participated more actively and even took on additional missions. However, some participants found even the basic mission of wearing the activity tracker every morning challenging and dropped out of the project. (P6:12).*

The RN-PHC highlighted that certain older adults required more time to acclimate to the devices and faced challenges in adapting to AI·IoT projects. However, they emphasized the significance of delivering services to vulnerable older adults and expressed confidence that addressing technical issues would enable effective service provision. In essence, they believed that with adequate education, vulnerable older adults would be able to access the services they require.

*It’s taking a while for our participants to adapt to the devices. We found that there were only a few people who were suitable for the IOT project among the home visiting project participants. (P9:2).*

*In my opinion, we should provide assistance to vulnerable individuals. With solutions for mobile phone compatibility issues and adequate education, I think the IOT project can work for older adults and vulnerable participants (with help from their children if needed). (7,34).*

## 4. Discussion

This study aims to explore the facilitators and barriers experienced by RN-PHCs during the implementation of AI·IoT-PP in 2021, with a primary focus on understanding the implications of integrating this emerging technology into home-visiting healthcare services. This study emphasizes the effective use of AI-IoT-PP in enhancing public health care for older adults, thanks to goal-setting functionalities and government support.

Our study highlights the transformative potential of digital health technologies, especially AI·IoT-PP interventions, in enabling older adults to actively manage their health. The pilot phase of the AI·IoT-PP intervention proved feasible and was positively received, demonstrating the effectiveness of mobile technology in enhancing communication and goal setting between RN-PHCs and clients. These findings echo community-based studies that underscored the critical role of health worker involvement and efficient clinician workflows in the successful adoption of mobile apps among low-income populations and vulnerable families (9, 14). This aligns with the literature (14, 15), which advocated for mHealth applications as significant facilitators in health management. Mohammed’s study showcased how mHealth apps can assist individuals with chronic conditions in effectively tracking their health-related goals, indicating a potential for these apps to boost health-promoting behaviors. Our findings further reaffirm the necessity of health worker participation in ensuring successful mobile app adoption within disadvantaged populations. The RN-PHCs were instrumental in implementing a daily digital health program that delivered high client satisfaction, leveraging AI·IoT-PP interventions to improve communication, goal setting, and provide user-friendly access to health features. In particular, the goal-setting functionality emerged as critical for helping older adults set and attain personal health objectives, demonstrating how technology can encourage self-care and consistent progress in health management. Moreover, RN-PHCs reported that AI·IoT-PP interventions enabled timely interventions, leading to positive health behavior changes, with optimism regarding the program’s long-term outcomes. Second theme was Government Support for Digital Health Management. Given the nature of community-based health nursing, a limited number of RN-PHC are responsible for a large number of community-dwelling clients. Consequently, existing services have primarily focused on one-way interventions, including health screening, health and lifestyle counseling, referrals, and documentation (16). As part of the AI·IoT-PP, participants were equipped with health-related devices such as blood pressure monitors, blood glucose meters, pedometers, and smart scales. RN-PHCs reported that clients’ heightened satisfaction stemmed from receiving these devices free of charge and monetary incentives, which was made possible, in part, by government-funded support. Almost all RN-PHCs emphasized the indispensability of government support for the continued provision of services. This support is crucial not only for enrolling more clients and delivering services, as previously suggested (10), but also because of concerns regarding the program’s sustainability resulting from insufficient funding transparency. If the feasibility and effectiveness of the pilot project are confirmed, further analysis of the cost-effectiveness and suitability of government support should be conducted. Additional deliberation is necessary regarding the process of selecting appropriate candidates for AI·IoT projects among the recipients of home healthcare services.

The third theme identified in this study was the Tech Challenges, which served as barriers to Digital Health adoption among older adults. These difficulties encompassed technical glitches, usability issues with software, and connectivity problems, all of which obstructed the successful integration of these technologies, despite their immense potential. Existing literature echoes these challenges, indicating the widespread nature of these technical hurdles when older adults interact with digital health technologies (14, 17). Further complicating this issue, not all available telehealth technologies are suitable for older adults due to age-related changes like diminished vision, impaired hearing, and reduced dexterity, potentially restricting their ability to efficiently use various telehealth devices (18, 19). This study was unable to conclusively establish a direct correlation between the physical changes associated with aging in older adults, advanced age itself, and the technology challenges encountered. Further research is warranted to elucidate this relationship more precisely.

Moreover, these issues were further complicated by participant attrition and a lack of clear protocols for withdrawal, thereby underlining the pressing need for increased technical assistance, resources, and strategic planning for successful and sustainable adoption within this demographic (10). In response to these challenges, some studies have proposed potential solutions, such as the design of more user-friendly interfaces and the establishment of dedicated support hotlines to assist older adults in navigating these technologies (20).

Initially perceived as minor, these technical difficulties morph into intricate and disruptive obstacles during the implementation of interventions, requiring the addressing of additional barriers, including the capacity and integration of workflows for RN-PHC. As part of their role, RN-PHCs are tasked with providing technical support to clients, which necessitates possessing the necessary skills and familiarity with the application while promptly resolving client concerns.

Despite the profound influence of information technology in driving digitization in daily life, a persistent disparity between those who have access to information and those who do not remains. Consequently, addressing this information gap among vulnerable populations has emerged as a pressing concern (21). Given this context, the incorporation of previous literature on similar technical challenges and their potential solutions provides a more holistic understanding of the complexities involved in adopting digital health technologies, especially among older adults. The digital alienation of vulnerable older adults, particularly those living alone or with diminished cognitive abilities, is exemplified by their challenges in using mobile phones, facing connectivity issues, and even lack of smartphone ownership, necessitating comprehensive education and support. However, RN-PHC highlighted that with time and adequate education, these vulnerable groups could successfully adapt to AI-IoT projects and gain access to essential services.

South Korea has the highest smartphone distribution rate globally. However, the level of digital health literacy varies significantly depending on age and income levels. Almost all individuals in their 20s and 40s own smartphones, while the ownership rate decreases to 80% for those in their 60s and is as low as 38% for individuals aged 70 and above (22). In the context of a globally aging population, it is crucial to engage older adults in digital technology, including mHealth, to promote their health and functioning (23). The literature suggests that telehealth and mHealth offer valuable solutions for remote support to frail older adults (24, 25).

However, the older adult population in particular, frequently face exclusion and a sense of alienation from utilizing information technology. This phenomenon, known as digital alienation, often leads to feelings of unfamiliarity and helplessness due to a widening technology gap (26–28). Previous research demonstrated that individuals with higher income and educational levels are more inclined to adopt and express satisfaction with technology, whereas those with lower income and educational levels experience greater digital alienation (28). Considering that recipients of community-based home healthcare services often belong to low-income groups with limited educational backgrounds, it is crucial to enhance their confidence and competence in using technology. Interventions incorporating video technologies and telephone support have shown promise in reducing isolation and improving health outcomes (4, 21). As frontline service providers, RN-PHCs play vital roles in endorsing and adopting technological solutions. Their adoption of technology enhances client engagement, satisfaction, and overall outcomes (9, 29). Therefore, for participants experiencing exclusion and a sense of alienation, a variety of approaches including video technologies, telephone support, or a combination of online and offline visits could potentially be beneficial.

In the era of the digitalization and smartification of nursing practice, nurses are required to act as catalysts for change and possess the ability to seamlessly deliver advanced nursing services. As society increasingly embraces contactless practices, there will be a growing demand for various forms of nursing services. Hence, it is essential to develop and provide patient-centered, tailored nursing education, counseling, and care management programs. These programs should utilize blended approaches that combine online services with traditional face-to-face consultation and education. Amidst these transformative changes, it is of utmost importance for RN-PHC to recognize the unique needs of vulnerable populations and assume an even more significant role in ensuring that their health is effectively managed without facing marginalization.

### 4.1. Limitation

This study acknowledges a few limitations. Initially, as it was a pilot study, the AI-IoT-PP was deployed in a restricted number of health centers, potentially limiting the wide-ranging applicability of our results. Despite the lingering potential for either over- or underestimation, we believe the preservation of an open and non-judgmental interview is key to accurately depicting participants’ experiences and viewpoints. While we do not foresee major issues regarding the authenticity of their responses, the possibility of overestimation or underestimation, given the RN-PHCs’ role in implementing the pilot program, is a consideration that we have recognized. Despite efforts, the complete eradication of these biases remains a challenge due to sample size restrictions. An additional limitation lies in the relatively low adoption rate of AI·IoT-PP among enrollees of the home-visiting healthcare service, even with explicit registration suggestions. This pattern underscores potential hurdles in promoting the wider adoption of AI·IoT-PP within this demographic. To address these limitations, we understand the imperative for more extensive research, particularly emphasizing the verification of the authenticity and accuracy of data obtained from RN-PHCs.

## 5. Conclusion

This study underlines the transformative potential of the AI-IoT-PP in older adults’ healthcare in the public health sector. Key facilitators include technology-assisted behavioral adoption, real-time interventions, and government support, which are particularly relevant during the COVID-19 pandemic. However, technological hurdles and disparities in digital literacy skills among the older adults, especially those who are economically and educationally disadvantaged, have emerged as significant barriers. Therefore, strategies aimed at enhancing digital literacy and addressing technological challenges are critical for ensuring a more inclusive and effective healthcare system.

While these challenges persist, this study also reveals that with continued support and training, the older adult population can adapt to healthcare technology. These findings reinforce the necessity for persistent efforts to support this demographic in an evolving digital healthcare landscape. Hence, this research underscores the significance of technological interventions, such as AI·IoT-PP, especially for vulnerable demographics in the public health sector. This further highlights the necessity for all-encompassing strategies to optimize their effectiveness while maintaining an inclusive approach.

## Data availability statement

The original contributions presented in the study are included in the article/supplementary material, further inquiries can be directed to the corresponding author.

## Ethics statement

The studies involving human participants were reviewed and approved by Ajou University IRB. The patients/participants provided their written informed consent to participate in this study.

## Author contributions

HO conceived and designed the study, conducted data collection (interviews) performed data analysis, and contributed to the interpretation of the findings. SB conceived and designed the study, participated in data collection (interviews, member checks), contributed to the data analysis process, and played a role in the interpretation of the findings. SB and HO contributed to the writing and revision of the manuscript. All authors contributed to the article and approved the submitted version.
